# Supplementary material for: Metasurface magnetless specular isolator
Source: Sci Rep. 2022 Apr 5;12:5652. doi: 10.1038/s41598-022-09576-0 (PMC8983700; doi:10.1038/s41598-022-09576-0)
Supplement: Supplementary file 1 — Supplementary Information. [file 41598_2022_9576_MOESM1_ESM.pdf]

Supplementary Material

Figure 1 shows that the frequency responses of the reflection coefficients in the opposite two directions are perfectly symmetric, as expected for simple Lorentz resonances, but different from each other, the absorption-direction response being much narrower than the passing-direction response.

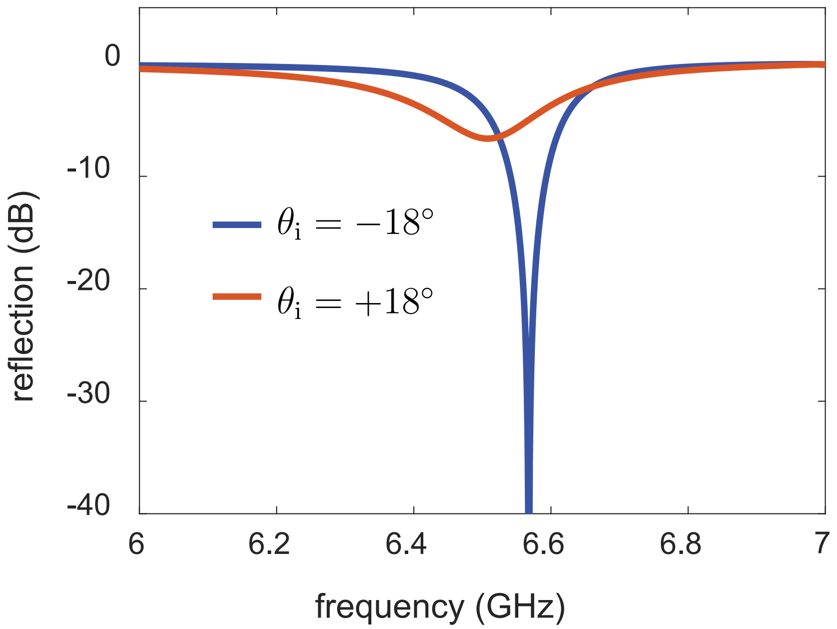

Figure 1: Full-wave simulation of the reflection coefficient versus frequency for $\theta_i = -18°$ and $\theta_i = +18°$. The two frequency responses are symmetric but different due to the nonreciprocal response of the transistor-loaded metaparticle.
